# Stability of transversal correction with hybrid maxillary expansion appliance in bone and tegumental piriformis opening in relation to bone age and maturation of the midpalatal suture

**DOI:** 10.4317/jced.59575

**Published:** 2022-05-01

**Authors:** Vandressa de Marco, Karina-Maria-Salvatore Freitas, Renata-Cristina-Faria-Ribeiro de Castro

**Affiliations:** 1 DDS, MSc. São Leopoldo Mandic Dentistry Faculty, Campinas, Brazil; 2 DDS, MSc, PhD. Professor, Department of Orthodontics, Ingá University Center Uningá, Maringá, Brazil; 3 DDS, MSc, PhD. Professor, Department of Orthodontics, São Leopoldo Mandic Dentistry Faculty, Campinas, Brazil

## Abstract

**Background:**

To evaluate the stability of the transverse correction with a hybrid maxillary expansion appliance in the bone and tegumental piriformis opening in relation to bone age and maturation of the midpalatal suture (MPS).

**Material and Methods:**

15 patients with a mean initial age of 14.9 years (SD=1.50), 7 (46.7%) were female and 8 (53.3%) were male, treated with a hybrid maxillary expander. Cone beam computed tomographic (CBCT) images were collected in three phases: T1 (orthodontic records), T2 (21.33 days (SD=10.68) after the end of expansion screw activation) and T3 after 9.13 months (DP=2.41) after the expansion screw was activated. In CBCT, measurements were performed in the nasal cavity considering the tegumental piriform opening (sagittal-axial sections) and bone (sagittal-axial-coronal sections) and the stage of MPS maturation (sagittal-axial sections). Repeated measures ANOVA was used for continuous variables and Friedman’s ANOVA for the ordinal variable followed by Bonferroni’s tests for *p*<0.001, in relation to time.

**Results:**

There were significant differences between T1 and T2 (*p*=0.041), between T2 and T3 (*p*<0.001) and between T1 and T3 (*p*=0.041). Regarding bone age by cervical vertebrae maturation, 20% were in stage CS3, 40% in stage CS4, 26.7% in stage CS5 and 13.3% in stage CS6. There was a significant increase in tegumental piriformis opening between T1 (M=32.19, SD=3.79) and T2 (M=34.82, SD=2.81) (*p*=0.008), followed by a significant decrease in T3 (M=34.64, SD=2.73) (*p*=0.021), as well as in the opening of the bone piriform, between T1 (M=21.30, SD=2.47) and T2 (M=25.35, SD=2.21) (*p*<0.001), followed by a significant decrease in T3 (M=24.89, SD =2.30) (*p*=0.018).

**Conclusions:**

The hybrid maxillary expansion appliance was effective in opening the midpalatal suture of all patients in the present study, without influence of the initial stage of MPS maturation and bone age. There was a relapse of the increase in the bone and tegumental piriform openings.

** Key words:**Maxillary expansion, orthodontic anchorage procedures, nose.

## Introduction

The evaluation of transverse skeletal changes of the maxilla after conventional rapid expansion (without bone anchorage), in a hybrid (with bone anchorage) and surgical form and its effects on the airways and nasal cavity in the short, medium and long term, is not actual (1,2). Maxillary atresia should be treated as soon as possible to encourage correct growth of the maxillomandibular complex (3,4). The first choice of orthodontic treatment for this skeletal problem is the use of an expander, promoting forces on the bony palate to open the Midpalatal Suture (MPS), and promote maximum bone repositioning, with minimal dentoalveolar effects (5). Copello *et al*. (6) reported that maxillary expansion supported by mini-implants was an effective alternative with regard to orthopedic changes, avoiding undesirable effects in late adolescence and adulthood. The most recent studies have shown better results with hybrid expanders when compared to conventional ones, even in mature patients (4,7,8). Coloccia *et al*. (4) reported that the hybrid expansion showed no undesirable effects on dentoalveolar expansion in late adolescents, being an option to surgical expansion. Copello *et al*. (6), in a systematic review and meta-analysis confirmed that the use of hybrid expanders could reduce the loss of buccal bone when compared to the conventional one.

Badreddine *et al*. (9) evaluated in the short term (3 months after expansion) the effects of Rapid Maxillary Expansion (RME) in relation to the skeletal and tegumental structures of the nose in 55 patients with maxillary atresia, based on computed tomography at two different times, divided into a control group and one submitted to RME. In the group that underwent expansion, there were significant increases in all skeletal and tegumental variables (*p*>0.05), and when comparing the groups, the change with the greatest increase occurred in the piriform opening after expansion (*p*=0.001).

Bazargani *et al*. (1) in their randomized study, aimed to compare the skeletal and dentoalveolar effects after conventional and mini-implant-supported RME, evaluated from cone-beam computed tomography (CBCT), with a sample of 52 patients, divided into two randomized groups. CBCT pretreatment and one year after treatment were used. The group with skeletal anchorage had a significantly greater maxillary expansion than the conventional group. This can also be observed in relation to the increase in the nasal cavity, in the anterior portion, and the expansion was almost twice greater in the group with skeletal anchorage, remaining significantly greater one year after expansion. However, there was no difference in stability one year after treatment.

There is a scarcity of literature regarding the stability of the transverse correction with a hybrid maxillary expansion appliance in the bone and tegumental piriformis opening in relation to bone age and maturation of the MPS. It is expected, with this study, to bring answers regarding the stability of the achieved after opening along the midpalatal suture in the bone and tegumental piriformis opening and to demystify the influence of the initial maturation of the MPS and vertebral bone maturation in young adult patients.

## Material and Methods

This is a longitudinal clinical study based on the analysis of maxillary CBCT scans from the database of a Private Clinic (RCFRC) located in the city of Campinas-SP, Brazil, approved by the Ethics and Research Committee

of the São Leopoldo Mandic Faculty (number 32707520.0.0000.5374, decision 4.204.616).

The sample size calculation was performed with the aim of comparing quantitative variables (Bone and Tegumental Piriform Opening) at 3 moments: T1 (before the expansion appliance was installed), T2 (after the end of activation of the expander screw) and T3 (after 8 months of bone healing). Calculations performed with the G*Power program (10) lead to the conclusion that a sample of 15 patients allows detecting moderate effects (f=0.25) with repeated measures ANOVA, with a test power of 80% and a significance level of 5%. The sample consisted of 15 patients (13 to 17 years old), with a mean age of 14.9 years (SD=1.5) at T1. Seven (46.7%) were female and 8 (53.3%) were male. As for the vertebra stage, 3 (20%) were in the CS3 stage, 6 (40%) in the CS4 stage, 4 (26.7%) in the CS5 stage and 2 (13.3%) in the CS6 stage. Regarding the MPS Maturation Stage, at T1, 3 (20.0%) patients were in Stage A, 4 (26.7%) in Stage B, 6 (40.0%) in Stage C and 2 (13.3%) in Stage D (11,12).

**Figure 1:**
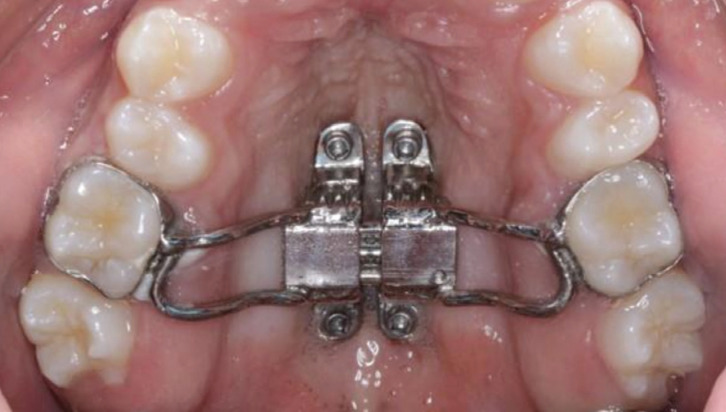
Hybrid Hyrax Appliance.

Each patient underwent a standardized protocol, and received a maxillary expander (Fig. 1) that was anchored in the bony palate, due to the presence of more atresic palates, using four mini-implants (13) that were supported in bands on the maxillary first molars, installed bilaterally between the projections of the first permanent molars and second premolars at 3mm from the MPS, so that they remain parallel to the MPS (14,15).

Inclusion criteria were: patients without previous orthodontic treatment; Complete permanent denture up to second molars; maxillary atresia evaluated clinically with or without unilateral or bilateral posterior crossbite; Minimum age between 13 years and maximum of 18 years; Treated with the same protocol of maxillary expansion supported by 4 mini-implants (Hyrax hybrid expander) (Fig. 1); same type of expander screw and insertion site in the posterior region of the palate; complete medical records in the 3 phases studied. The expansion screw was activated for 14 days, after which the hybrid expansion appliance remained as retention for 8 months.

The evaluation of the degree of MPS maturation was performed from the CBCT using the OP300 Maximus device (FOV 8x8; 90Kv, 6.3mA and 4.5 mAs, voxel 0.25) with the visualization of the DICOM file, in axial, coronal and sagittal sections. Measurements were performed using the Blue Sky Plan® software.

In the sagittal view, the patient’s head was adjusted so that the anteroposterior axis of the palate is horizontal, oriented in relation to the Frankfurt plane and the midsagittal plane (9,11).

The cross-section was used to assess MPS maturation, the palate being parallel to the horizontal line of the software used. After its insertion along the palate, the most central transverse slice in the superior-inferior dimension (nasal to oral surface) is used to classify the maturation stage of the MPS (12).

-Measurements

Alterations promoted in the anterior region of the nasal cavity:

A. The bone and tegumental piriform opening: the most anterior bone opening of the nasal cavity, with limits on the upper nasal bones and the maxillary nasal processes laterally (9).

B. Opening of the MPS: performed after expansion, with reference to the region of the mini-implants in the anterior and posterior regions, to assess the opening at the T2 CBCT (16).

**Figure 2:**
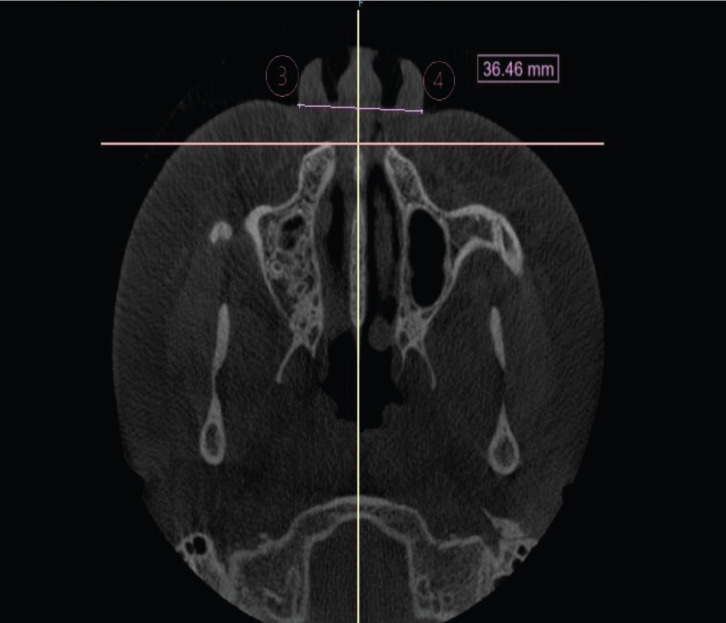
Measurement of the tegumental piriform opening.

The size of the piriform tegumental opening was obtained in the axial section by the linear distance (mm) between the points Right Alar (Acr) and Left (Acl) Curvature. The size of the piriform bone aperture was measured in the coronal section by the linear distance (mm) between the superior and inferior tangents of this structure above the midsagittal plane, (Fig. 2).

-Analysis of cervical vertebrae maturation

Bone age was evaluated using the Vertebral Maturation Index (VMI) adapted by Baccetti *et al*. (17) through the analysis of sagittal slices of CBCT images to classify the cervical vertebrae according to the body morphology of C3 and C4 and the formation of concavity at the lower edge of C2, C3 and C4. The classification nomenclature was slightly modified, instead of letters, the different stages were described with numbers from 1 (I) to 5 (V).

-Analysis of the maturation of the median palatine suture (SPM)

**Figure 3:**
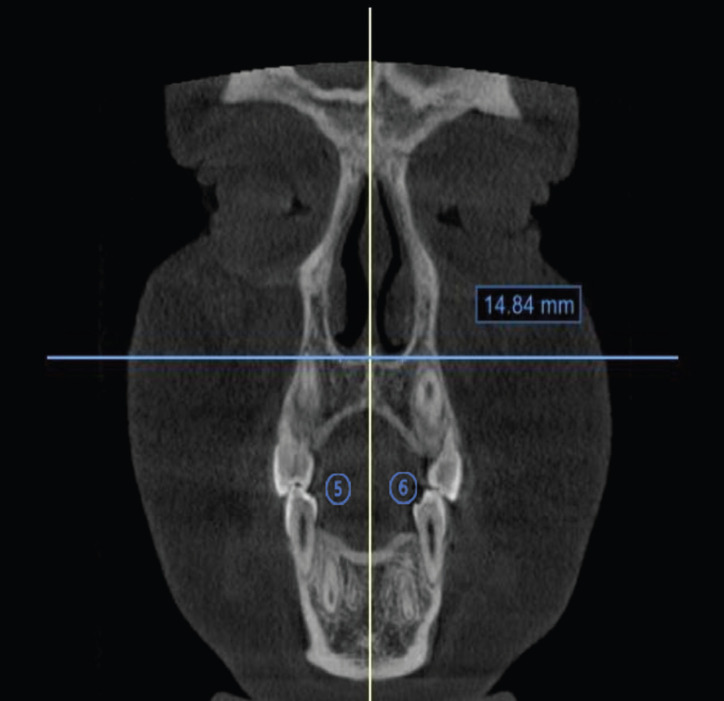
5 Left Tangent Line to the MSP. 6 Right Tangent Line to the MSP. Location of points in the coronal view for measuring the bony piriform opening.

Initially, the standardization of the head position was performed, the measurement of the MPS maturation stage was started, evaluated from the images of axial slices of the MPS region in the CBCT images using the Ok *et al*. (18) method; instead of letters, the different stages were described with numbers from 1 (A) to 5 (E), in which stage 1 to MPS appears as an almost straight line, with high radiopaque density, with little or no interdigitation and in the stage 5 there is fusion of the MPS in the region of the maxillary bone, the bone density is the same as the adjacent palatine bone, and it is not possible to visualize the suture, (Fig. 3).

-Statistical analysis 

Statistical data analysis was performed using the Statistical Package for the Social Sciences (SPSS) program, version 26 for Windows (IBM Corp. Released, 2018). The normality of the bone and tegumental piriform opening variables was tested and validated with the Shapiro-Wilk Test. The study of the measurement error of the continuous variables (bone and tegumental piriform opening) was carried out using the paired t tests and the Intraclass Correlation Coefficient (ICC). As for the Stage of Maturation of the MPS and the opening of the MPS (categorical variables), the percentages of agreement between the initial classification and the repetition were calculated and the Kappa Coefficient was applied. To respond to the research objectives, for the continuous variables (bone and tegumental piriform opening) repeated measures ANOVA was used to assess the significance of differences over time. To identify the times with significant differences, multiple comparison tests were performed (T1-T2, T2-T3 and T1-T3) and MPS Maturation Stage was used Friedman ANOVA by the Bonferroni Method. For the study of the correlation between the alterations of the Bone and Tegumental Piriform Opening, Stage of Maturation of the Midpalatal Suture and the Stage of the Vertebrae, the Spearman Correlation Coefficient was used. A significance level of 5% was considered.

## Results

-MPS Maturation Stage

At T1, 3 (20%) patients were in Stage A, 4 (26.7%) in B, 6 (40%) in C and 2 (13.3%) in D. At T2, the 15 patients were all in Stage A. At T3, none were observed in Stages A or B, 5 (33.3%) were in Stage C, 9 (60.0%) were in Stage D, and 1 (6.7%) were in Stage E. The Friedman ANOVA results (*p*<0.001) and multiple comparison tests with Bonferroni correction showed that the differences were significant between T1 and T2 (*p*=0.041), between T2 and T3 (*p*<0.001) and between T1 and T3 (*p*=0.041).

-Relation of the MPS Maturation Stage with the Vertebra Stage (T1)

The correlations of the T1 Vertebrae Stage with the MPS Maturation Stage at T1 (R= -0.203, *p*=0.468) and at T3 (R= -0.199, *p*=0.478) were negative but not significant.

-Relationship between Bone and Tegumental Piriform opening at T1, T2 and T3

In the tegumental Piriform opening, there was a significant increase in the mean between T1 (M=32.19, SD=3.79) and T2 (M=34.82, SD=2.81) (*p*=0.008), followed by a significant decrease in T3 (M= 34.64, SD=2.73) (*p*=0.021). The mean value recorded at T3 was significantly higher than that recorded at T1 (*p*=0.011). Similarly, in the evolution of the mean Bone Piriform opening there was a significant increase between T1 (M=21.30, SD=2.47) and T2 (M=25.35, SD=2.21) (*p*<0.001), followed by a decrease in T3 (M=24.89, SD=2.30) (*p*=0.018), with the mean value in T3 significantly higher than that recorded in T1 (*p*=0.001) as shown in Table 1.

**Table 1:**
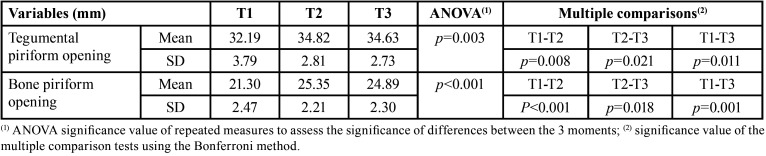
Characterization and comparison of tegumental piriform opening (mm) and bone piriform opening (mm) between T1, T2 and T3 (N=15).

The comparison of the tegumental piriform opening in relation to the bone piriform opening showed a significant increase between T1 and T2 phases (*p*=0.008), remaining stable in the T2-T3 phase (*p*=0.021). The same occurred with the bone piriform opening, with an increase from 21.30 to 25.35 mm (*p*<0.001), remaining sTable in the T3 phase (*p*=0.018).

-Relationship between Bone and Tegumental Piriform Opening x Vertebrae Stage and MPS Maturation Stage (T1)

The results of the study of the correlation of the alterations of the Tegumental Piriform opening and of the Bone *Pi*riform opening (difference between T1 and T2, difference between T1 and T3, difference between T2 and T3) with the initial Stage of MPS Maturation (T1) and with Stage of the Vertebra (T1) showed that none of the correlations was significantly different from zero (*p*>0.05). However, the negative correlations of changes in the Tegumental and Bone Piriform opening between T1 and T2 and between T1 and T3 with the Vertebra Stage (T1) and positive correlations with the Initial Stage of MPS Maturation (T1) are in Table 2.

**Table 2:**
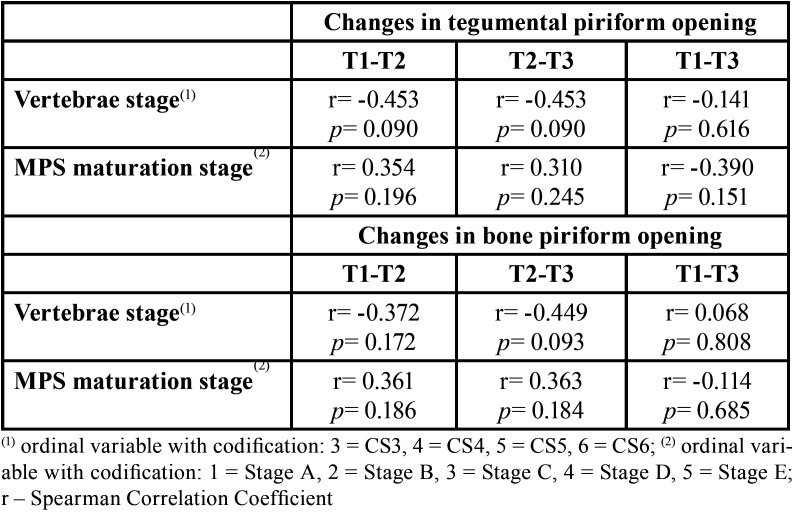
Correlation of changes in Tegumental Piriform opening and Bone Piriform opening with the Initial Stage of MPS Maturation (T1) and with the Vertebrae Stage (T1) (N=15).

## Discussion

It is not new that there is a consensus regarding the most effective method for diagnosing the degree of ossification of MPS, through CBCT, in adolescents, post-pubertal and adults (17,19). In this study we evaluated the stage of MPS maturation, at T1 20% of the patients were in stage A, 26.7% were in stage B, 40% in stage C and 13.3% in stage D. In T2, all patients had the sutures open in parallel, in stage A of the classification. Ngan *et al*. (20) investigated the skeletal response of using the hybrid expander appliance from CBCT, in patients with advanced CVM 4 or higher being characterized as skeletally mature and sutural ossification stages C, D and E as a diagnostic method. After expansion, suture rupture was observed in 100% of the patients, with a parallel MPS opening pattern in the coronal and axial planes. In T3, 33.3% of the patients were in stage C, while 60% were in stage D and only 6.7% in stage E, that is, after 08 months of retention there was a bone neoformation in the sutural region. Similar results were found in systematic reviews (1,21), which highlight articles that compare the effectiveness of using appliances with skeletal anchorage, which had minimal dental side effects and significant airway changes.

Regarding the bone age of the sample of the present study, 20% of the cases are in CS3, 40% in CS4, 26.7% in CS5 and 13.3% in CS6. Angelieri *et al*. (22) in an untreated cross-sectional sample, showed that in postpubertal patients (CS4 and CS5) 13.5% of patients were in stage CS5. In the present study, 40% of the patients in Stage C of MPS maturation were obtained at T1, followed by 26.7% in B, 20% in A and 13.3% in E. After the expansion in stage T2, the patients were with the parallel opening in MPS stage A, reporting that the appliance was effective in relation to the treatment of maxillary atresia. In T3, after 08 months, most patients (60%) had MPS in stage D, while 33.3% in C and only 6.7% in E, resulting in bone neoformation between MPS (23).

The Initial Stage of MPS Maturation and the Vertebral Stage at T1 showed that there was no significant correlation with the degree of total opening of the midpalatal suture in all patients in the present study sample at T2 phase. The bone age evaluated by the vertebrae does not significantly correlate with the bone and tegumental piriform opening between T1, on the other hand, there is a significant correlation between the degree of maturation of the MPS with the tegumental and bone increase of the piriform opening after hybrid maxillary expansion. The study by Jang *et al*. (24) with 99 patients and a mean age of 12.03 years did not corroborate the present study as it found a strong relationship (*p*<0.01), however, the study does not report on the duration of follow-up tomographic evaluation and does not mention the type of expander used. Other authors that corroborate the present study evaluated patients with a higher age group, Goruco-Coskuner *et al*. (25) 19-30 years (n=50) did not find a correlation between MPS opening and cervical bone age after treatment with hybrid maxillary expander (r=0.030; *p*=0.839). In our study, most of the patients were in stage C of MPS maturation with equal distribution between the MCV stages in CS3, CS4 and CS5 (r=-0.203, *p*=0.468), as well as the patients in the Goruco-Coskuner *et al*. (25) study which were also in stage C of MPS and stage CS4 of vertebrae. The extent of MPS interdigitation is independent of bone age and chronological age, and the use of MCV stage should not be used for the diagnosis of MPS maturation, unlike what was proposed by Angelieri *et al*. (22) who suggested the diagnosis at from the anteroposterior features of MPS.

 Most articles and systematic reviews (1,26,27) which evaluated the skeletal and tegumental tissue in relation to maxillary expansion with a hybrid appliance, had the results evaluated in the short term, ranging from 3 months (2,7,28) to 6 months (26,27,29) on average, different from the present study that evaluated the changes in these structures in the long term with 8 months of retention. In these studies, the results showed that the changes that occurred in the MPS and in the anterior region of the nasal cavity were the result of the RME (9,30). The increase in bone and tegumental tissues occurred due to the absorption by these of the forces dissipated by the hybrid expander appliance, since the disjunction of the maxilla promotes the displacement of the maxillary bones laterally along the lateral walls of the nasal cavity, promoting an increase mainly in the nasal width in its anterior portion (31). In the present study, the mean time between T2 and T3 was 8 months, after the end of the expansion screw activation. There was stability of the increase in the bone and tegumental piriform opening in the T3 phase, on the other hand, other studies approach a mild relapse with the accommodation of the tegumental tissues, however, unlike the present study, the T3 phase was evaluated without the expander appliance and with a fixed corrective appliance, which may have affected this movement and indirectly caused a reduction in the width of the anterior region of the nasal cavity, associated with the elastic activity existing in both the palatal and tegumental soft tissues (32,33).

In this study, we found a significant increase in the bone piriform opening between T1 and T2, with a mean of T1 of 21.30 (sd=2.47), while T2 had a mean of 25.35 (sd=2.21). Similar results were obtained by Badreddine *et al*. (9) when exposed that the group undergoing RME showed a significant increase in the bone piriform opening, with a mean significant increase (*p*<0.001) of 1.98 mm. Bazargani *et al*. (1) corroborated the results of the increase in the nasal cavity, in the anterior portion, the expansion was almost twice as greater in the group with skeletal anchorage when compared to the conventional one. Regarding the evaluation between T2 and T3, there was a significant reduction, with the average of T2 being 25.35 (sd=2.21), while T3 had an average of 24.89 (sd=2.30) (*p*=0.018). Yi *et al*. (2) evaluated from CBCT the changes in mini-implant-assisted maxillary expansion and airway structures in 19 patients aged between 15 and 29 years. CBCT was evaluated before installing the appliance and 3 months after completion of the expansion. Measurements were performed to assess the entirety of the skeletal base and airway expansion. This study observed that the anterior width of the nasal cavity had significant changes of 1.63 mm (*p*<0.01) in the short term and with the use of an expander only supported by mini-implants in the palate. The only study that evaluated nasal alterations in the long term was Bazargani *et al*. (1) observed after one year the alterations promoted by RME remained close to the value found in the evaluation after expansion, when evaluating 52 patients. The sample was divided into two groups: one with the conventional expander appliance (supported on the first permanent molar) and other with the hybrid expander appliance (supported on the first permanent molar and two mini-implants in the anterior region of the palate). Both groups received 0.5mm/day expansion screw activation and were removed after a 6-month retention period. Regarding the nasal structure, in the group with the conventional appliance there was an increase of 2.6 mm between T0 and T1, while for the group with the hybrid expander this increase was 3.3 mm (*p*=0.069). After 1 year, the group with the conventional appliance had a slight relapse of 1.2 mm, as well as in the group with the hybrid expander, with a relapse of 1 mm (*p*=0.025). Long-term evaluation revealed that MPS bone expansion was greater in the hybrid appliance group, however clinically not significant and treatment stability after 1 year of treatment was similar in both groups.

In the present study, the increase in tegumental alterations of the piriform opening on T1 to T2 (*p*=0.008) remained sTable in the long term. Similar results were observed in Badreddine *et al*. (9) with an increase of 1.13 mm, with statistical significance (*p*<0.001). For Lee *et al*. (34) in a sample of 30 patients with a mean age of 20.46 years, the tegumental piriform opening had a significant increase after RME, with an increase of 1.2 mm between T0 and T1 (*p*<0 .01), but without evaluation after the MPS bone neoformation period. Lim *et al*. (32) evaluated 24 patients with a mean age of 21.6 years who underwent maxillary expansion with a hybrid expander appliance, investigating the transverse changes and their stability after 1 year of treatment, in which there was a significant increase in the nasal floor and cavity after expansion with a hybrid expander appliance, during T0 (before expansion) -T1 (one month after expansion) with an increase of 1.61 mm (*p*<0.001). However, in the evaluation from T1 to T2 (one year after expansion), this structure showed a reduction of 0.37mm (*p*<0.0001) when compared to the initial measurement.

The effects of the hybrid expander appliance are not limited only to the maxilla with the opening of the MPS, because when this separation occurs, the intermaxillary suture opens and, consequently, an increase in the bone and tegumental piriform opening occurs. If the period of bone neoformation is respected, this separation is filled by new bone that tends to remain sTable, as we found in the present study sample. The degree of success of opening the MPS in young adults is related to the initial maturation of the MPS, on the other hand the maturation of bone age by the vertebrae has no significant correlation.

Although we do not have a control group with other treatment protocols or age, in the course of this discussion it is observed that the results of this study corroborate the literature that also finds better results in bone opening with hybrid maxillary expanders and no correlation with age chronology, as observed in the study by Goruco-Coskuner *et al*. (25).

## Conclusions

Treatment with a hybrid maxillary expander appliance resulted in the opening of the midpalatal suture and an increase in the bone and tegumental piriform opening.

The increase in the bone and tegumental piriform opening remained stable 8 months after the end of the screw activation of the hybrid maxillary expander appliance.

There was a correlation between the initial maturation of the midpalatal suture with the increase in the bone and tegumental piriform opening and no correlation with bone age.
